# The Effect of Deworming School Children on Anemia Prevalence: A Systematic Review and Meta-Analysis

**DOI:** 10.2174/1874434601812010155

**Published:** 2018-07-31

**Authors:** Tadele Girum, Abebaw Wasie

**Affiliations:** * Department of Public health, college of Medicine and Health Sciences, Wolkite University, Wolkite City, Ethiopia

**Keywords:** Deworming, Albendazole, Anemia, Haemoglobin, School health, Parasite infection

## Abstract

**Introduction:**

High prevalence of anemia attributable to intestinal parasite infection occurs among children in developing countries. As a result mass treatment of all children with anti-helminthic drugs particularly in school setting is being implemented. There are few studies conducted to assess impact of deworming on anemia prevalence among school children with inconclusive finding. Therefore we aimed to conduct a systematic review on impact assessment of deworming on anemia prevalence or hemoglobin level of school children so that policy makers and other stalk holders could have pooled evidence on the direction to make decision.

**Methods:**

The review was conducted through a systematic literature search of articles published between 1998 and 2015. Five bibliographic databases and libraries: PubMed/Medline, Global Health Database, Embase, the Cochrane Library, and African Index Medicus were used. After cleaning and sorting, analysis was performed using STATA version 11. The pooled estimate was through a fixed-effects model. Heterogeneity was assessed by the I^2^ and publication bias through funnel plot.

**Results:**

Eight studies were retained for final analysis which enrolled a total of 1,005,239 school children. The overall change in the hemoglobin level after deworming was 1.62(95%CI=1.01-2.25) gram/deciliter. There was no difference between the random effect model and the fixed effect model. The prevalence of anemia was markedly changed after the program, particularly in the studies which implemented deworming with hygiene program, co-administration of iron and retinol.

**Conclusion and Recommendation:**

School based deworming program decreases prevalence of anemia and will contribute to reduction of anemia in the community. Therefore the program should be expanded in all areas and integrated with other child care programs.

## 1 INTRODUCTION

Chronic soil transmitted helminthic infections results in mal-absorption of macro and micronutrients which results in malnutrition like iron deficiency anemia [1]. Anemia is an overarching public health problem with wide range of consequences. It is the underlying and immediate cause for considerable number of maternal and child morbidity and mortality, loss of productivity among adults, impaired physical and mental development among children [2, 3]. The causes of anemia are multifactorial including diet, infection and genetics. High prevalence of anemia attributable to intestinal parasite infection occurs among children in developing countries [4].

Worms infect more than one third of the world’s population, with the most intense infections in children and the poor. In the poorest countries, children are likely to be infected from the time they stop breast-feeding, and to be continually infected and re-infected for the rest of their lives [5, 6]. As a result of the aforementioned fact World Health Organization (WHO) recommends mass treatment of all children with anti-helminthic drugs besides to provision of safe water, improving sanitation and health education [5, 7].

The most commonly used drugs for the deworming of common intestinal worm is albendazole (400 mg) or mebendazole (500 mg). They are administered as a single tablet to all children, regardless of size or age. One pill can cost as little as US$0.02 and only in the most highly infected communities is treatment required more than once a year [8].

The school setting offers an ideal distribution system for public health interventions of many types of health care**,** including health education, iron supplementation, and treatment or prevention of parasitic infections. The school years are an opportune time to intervene, and interventions must be based on sound epidemiologic understanding of the problem in this age group [4].

The need for program impact (Deworming) evaluation through epidemiologic survey is recommended by World Health Organization [8]. Accordingly there are few studies conducted to assess impact of deworming on anemia prevalence among school children in developing countries with inconclusive finding. Regular anthelmintic delivery (Deworming) has been shown in a number of studies to improve soil transmitted helminthes morbidity indicators, including growth, anemia, cognitive abilities and school attendance [3, 9, 10]. There are also other researches with finding of null impact of regular deworming on childhood anemia prevalence [11]. Therefore we aimed to conduct a systematic review on impact assessment of deworming on anemia prevalence or hemoglobin level of school children so that policy makers and other stalk holders could have pooled evidence on the direction program impact to make decision on the prospect of the program.

## 2 METHODS

### 2.1 Literature Search Strategy and Eligibility Criteria

This systematic review was conducted through a systematic literature search of articles published between 1998 and 2015 on the effect of deworming school children on anemia prevalence globally. Five bibliographic databases and libraries: PubMed/Medline, Global Health Database, Embase, the Cochrane Library, and African Index Medicus were used. Key words from Medical Subject Headings (MeSHs): Deworming, Albendazole, Mebendazole, Prazequantel, Anemia, Haemoglobin and Heamatocrit were used. In addition the references of each primary researches and reviews were screened and citations were uploaded into an EndNote XI library (EndNote, Carlsbad, CA, USA) and checked for duplications.

Selection was limited to research published in English language that reported the effect of school deworming program in anemia prevalence or hemoglobin level among school age children. Also Studies were eligible regardless of design and setting provided that they fulfill the inclusion criteria. The studies should be conducted on school based deworming program with a follow up period of at least two round administration of any of the medication recommended for deworming programs. The outcome of interest was level of hemoglobin or status of anemia among children who were dewormed one or more rounds. Studies conducted in the same location during the same time period were considered as potential duplicates and therefore excluded from the analysis.

### 2.2 Data Extraction and Abstraction

All studies we found on the electronic data base search were filtered for potential eligibility and duplications by preformed database and inclusion criteria. Only full studies which were retrieved from the abstracts were reviewed. If needed, and wherever possible, the authors were contacted for clarifications. From each eligible research, the following information was extracted: about author, study participants, studies (study design, cohort size, and setting), Type of medication provided, year of publication, year of study start and end, eligibility criteria, etc. All data were extracted independently and in duplicate using a standardized extraction form. Returned abstracts were reviewed and full texts retrieved if they contained relevant information. Meantime, each selected research was assessed for methodological quality and possibility of bias.

### 2.3 Data Analysis

After cleaning and sorting the final database was exported into Stata 11.0 for analysis (Stata, College Station, TX, USA) and the Analysis was performed using the ‘metan’ and related functions in STATA version 11 (College Station, TX). Each selected research was reviewed critically and assessed for effect. An outcome of interest was level of hemoglobin or status of anemia among school children who received deworming. Estimates were assessed for each study and standardized mean change with 95% confidence interval was used for level of anemia and its effect assessed for status of anemia. These were calculated with a fixed-effects model. Heterogeneity was assessed by the I^2^ and Bias was investigated by construction of funnel plots. Results were displayed visually in forest plots.

## 3 RESULTS

### 3.1 Studies Included

**Fig. (1):**
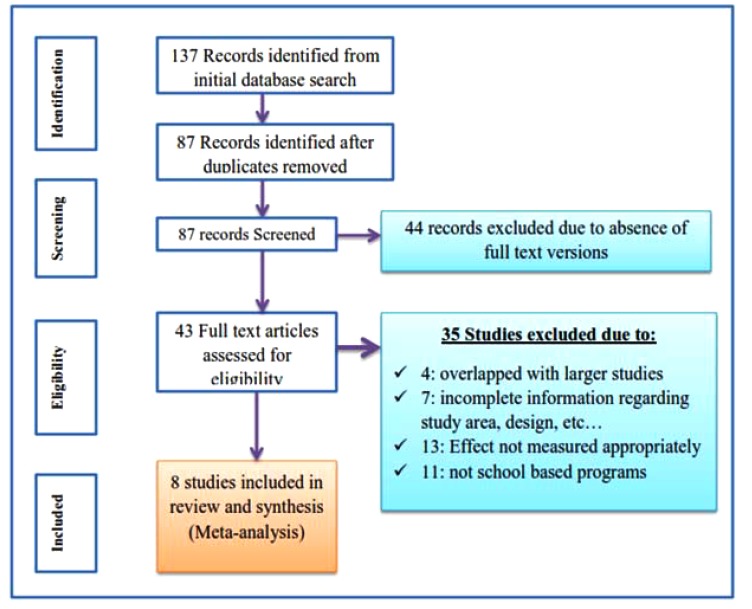
Flow chart for study search, selection and screening for the review.

 Initially the search identified 137 citations in the form of abstract, bibliography and full text research from the selected electronic data bases. All citations were transferred to preformed format of endnote and cleaned for duplications and 43 articles were identified for full text review. Of the 43 articles reviewed in full text, 8 articles were analyzed based on the inclusion and exclusion criteria and quality assessment. Among the identified 43 full text studies 35 were removed prior to analysis for different reasons: 4 over lapped with larger studies, 7 were incomplete in one of the inclusion criteria, 13 studies measured other effect of deworming other than anemia and hemoglobin level or measured inconsistently and 11 studies were removed due to program difference, in which they assessed among non-schooled programs (Fig. **1**).

### 3.2 Description of Findings

 The 8 full text studies that were retained for final analysis enrolled a total of 1,005,239 school children. Each study enrolled at least 144 school children with in the program and assessed the level of hemoglobin or status of anemia among dewormed and non-dewormed children. Children were received at least one or more round of deworming six months apart and followed for at least a year and the studies were published during the period 1998-2015. Of these studies 5 were cross-sectional and 3 were randomized control trials. Two studies assessed the effect of Mebendazole, 2 studies assessed the effect of Albendazole only and the rest four studies assessed the effect of Albendazole with Prazequantel administration. Four studies were conducted in Asia and four were from Africa. Survey characteristics are described in (Table **1**).

### 3.3 Pooled Estimates and Tests

**Fig. (2):**
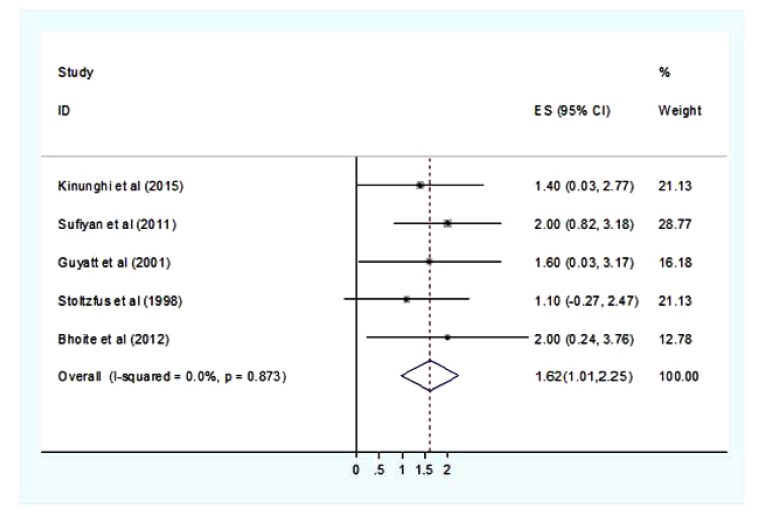
Forest plot showing change in hemoglobin level after deworming, weighted according to fixed-effects model.

 Heterogeneity tests showed no significant variations between studies (the weighted sum of squares on a standardized scale was not significantly different compared with expected weighted sum of squares) and I-squared showed that 0% of the observed dispersions are attributed to real rather than spurious variations. Also the funnel plot showed no evidence of publication bias. Therefore the fixed effect model was used to estimate the pooled increase in hemoglobin level after deworming. Based on the estimate, the overall change in the hemoglobin level after deworming was 1.62(95%CI=1.01-2.25) gram/deciliter. There is no difference between the random effect model and the fixed effect model (Fig. **2**).

The minimum change in hemoglobin after deworming of 1.1 (95%CI=0.27-2.47) gram/deciliter was observed in Stoltzfus *et al*. [17] report, while the maximum changes of 2 gram/deciliter was reported in two studies [14, 18]. However the variation was not statistically different between the studies. Besides hemoglobin change in some of the studies the prevalence of anemia was not changed even after repeated cycle of deworming. On the other hand the prevalence of anemia was markedly changed after the program in the studies which implemented deworming with hygiene program, co-administration of iron and retinol (Fig. **2**).

## 4 DISCUSSION

According to this systematic review school-based deworming program significantly increased the level of hemoglobin and reduced the burden of iron deficiency anemia. However the level of change depends on duration of the program or duration of follow up after administration of the drug, frequency of administration and integration of the deworming program with iron, retinol, nutrition and hygiene programs [12, 14, 16-18]. In all of the studies included in this review deworming caused improvements in iron status which is measured by protoporphyrin, a measure of iron-deficient erythropoiesis, and serum ferritin, a measure of iron stores and hemoglobin level. This is in line to previous reports [2-4, 9]. It is also long been known that deworming prevents hook worm infection which is one of the major cause of iron loss in children and in turn prevents anemia [5, 7, 8]

In studies which have longer follow up period and studies which integrated deworming program with iron supplementation the level of change in hemoglobin and reduction in the prevalence of anemia was higher. Among the studies included in the review Stoltzfus *et al*. [17] indicated that change in hemoglobin level and prevalence of anemia was observed on children who received two-three times a year. Similarly Bhoite *et al*. [18] and Sufiya *et al*. [14] reported that significant change in hemoglobin level was observed only when deworming was integrated with iron supplementation and hygiene education respectively. In line to our review significant increases in hemoglobin concentration were reported when deworming was provided along with iron supplementation among school children in south Africa [20], and India [21], and nutritional supplementation in Papua New Guinea [22].

However in some studies [13] despite improvements in iron status, the deworming program had no detectable effects on average hemoglobin concentrations or the prevalence of anemia. This may be due to the fact that change in the level of hemoglobin and prevalence of anemia takes a longer duration (12-15 months) after the child was dewormed [23]. As a result in some studies significant difference was not observed, when they have shorter follow up period. In line to this in another study, improvement in the hemoglobin level was not detected until 10 months of deworming intervention [18, 24]. It is expected that in our review too the positive impact of the study could be seen in the later years.

The primary aim of deworming is to prevent iron loss ascribed to hook worm infection. Therefore after deworming nutritional improvement and iron supplementation programs are compulsory for prevention of anemia [5-7]. Because deworming acts primarily by decreasing iron loss, in populations with limited absorbable dietary iron the expected effect from deworming would be to halt or slow the decline in iron status associated with hookworm infection.

In this regard, when children are not getting enough iron even though they are dewormed the hemoglobin level may not be changed. Particularly those children who are malnourished, absorption of iron will be reduced and change in hemoglobin may not be attained. On the other hand iron-specific indicators, ferritin and protoporphyrin which have a faster turnaround changed almost in all studies reviewed. Therefore, as observed in some other studies [17, 18] integration of deworming program with iron supplementation and nutrition programs yield better response.

## CONCLUSION AND RECOMMENDATION

School based deworming program (administration of intestinal anti-helminthic drugs) results in increase in hemoglobin level and decreases prevalence of anemia among school children. Thus school based intervention will contribute to reduction of anemia in the community. Better outcome was observed among programs which integrated deworming with iron and nutrition supplementation programs. Therefore school based deworming programs should be expanded in all areas and should be integrated with other child care programs.

## AVAILABILITY OF DATA AND MATERIALS

Please contact author for data requests.

## AUTHOR INFORMATION

TG: is Bsc/public health, MPH in Epidemiology and Biostatistics, Lecturer at Department of Public health, College of Medicine and Health Sciences, Wolkite University, Wolkite, Ethiopia

AW: is Bsc/public health, MPH in Reproductive health, Lecturer at Department of Public health, College of Medicine and Health Sciences, Wolkite University, Wolkite, Ethiopia

## Figures and Tables

**Table 1:** Characteristics of studies included in the review.

**Study**	**Year**	**Country**	**Design**	**SS**	**Drug Used**
[12] Kinugli *et al.*	2015	Tanzania	RCT	765	Prazeq & Albend.
[13] Awasthi *et al.*	2013	India	RCT	1000000	Albendazole
[14] Sufiya *et al.*	2011	Nigeria	Cross-sectional	301	Albendazole
[15] Watthanakalpich	2011	Thailand	Cross-sectional	239	Albendazole
[16] Guyatt *et al.*	2001	Tanzania	Cross-sectional	466	Prazq & Albend.
[17] Stoltzfus *et al.*	1998	Zanzibar	Cross-sectional	2924	Mebendazole
[18] Bhoite	2012	India	RCT	144	Albendazole
[19] Huong thi le	2007	Vietnam	Cross-sectional	400	Mebendazole

SS= Sample size, RCT= Randomized control trial, prazeq= prazequantel.
